# Effects of Physical Activity on Motor Development, Balance, and Psychosocial Well-Being in Children and Adolescents with Down Syndrome: A Systematic Review

**DOI:** 10.3390/jcm15176823

**Published:** 2026-09-03

**Authors:** Ignazio Leale, Manuel Gómez-López, Martina Macaluso, Daniela Smirni, Michele Roccella, Marianna Alesi, Giuseppe Battaglia

**Affiliations:** 1 Sport and Exercise Research Unit, Department of Psychology, Educational Sciences and Human Movement, University of Palermo, 90144 Palermo, Italy; 2 Department of Psychology, Educational Science and Human Movement, University of Palermo, 90128 Palermo, Italy; 3 Department of Physical Activity and Sport, Faculty of Sports Sciences, University of Murcia, Santiago de la Ribera, 30720 Murcia, Spain; mgomezlop@um.es; 4 Disability and Neurodiversity Center, University of Palermo (Ce.N.Dis.), 90133 Palermo, Italy

**Keywords:** exercise, motor skills, postural balance, quality of life, psychosocial functioning, child development

## Abstract

**Background:** Physical activity is relevant for physical and psychosocial development in children and adolescents with Down syndrome. However, evidence regarding the effects of structured physical activity interventions across different motor and psychosocial domains remains limited and heterogeneous. This systematic review aimed to examine the effects of structured physical activity interventions on motor and psychosocial outcomes in this population. **Methods:** The review was conducted according to PRISMA guidelines and registered in PROSPERO (CRD42025114068). Scopus, PubMed, and Web of Science databases were searched, with the final search conducted on 15 December 2025. Eligible studies were peer-reviewed articles published in English within the previous 10 years that investigated structured physical activity interventions in children and adolescents with Down syndrome and reported motor or psychosocial outcomes. Methodological quality was assessed using a modified Downs and Black Checklist. Due to substantial clinical and methodological heterogeneity across interventions, comparators, and outcome measures, findings were synthesized narratively. The certainty of evidence was assessed using the GRADE approach. **Results:** Five studies, including 186 participants, were included, of whom 166 had Down syndrome and 20 were typically developing children. The included studies reported improvements in motor skills, balance, coordination, and psychosocial outcomes. Interventions included Pilates, core stability and treadmill training, fundamental movement skills training, adapted football, and traditional Indian dance. Motor outcomes were generally associated with improvements in balance, postural control, coordination, and motor competence. Psychosocial outcomes were assessed in only two studies and showed preliminary improvements, including reductions in aggression, attention problems, anxiety/depression, and social problems. Overall, the certainty of evidence was rated as low across the assessed outcome domains. **Conclusions:** Structured physical activity interventions may be associated with improvements in motor development, while their effects on psychosocial outcomes appear encouraging but require further investigation. The findings should be interpreted cautiously because of the small number of studies, methodological limitations, and heterogeneity of interventions and outcome measures. Further high-quality randomized controlled trials with larger samples and standardized outcome measures are needed.

## 1. Introduction

### 1.1. Motor and Functional Characteristics

Down syndrome, first described in 1866, is one of the most common chromosomal abnormalities and the leading genetic cause of intellectual disability worldwide [1]. It is caused by the presence of an extra copy of chromosome 21, a condition known as trisomy 21 [2]. Individuals with Down syndrome exhibit characteristic brain and body features, including a smaller cerebrum and cerebellum, hypotonia, impaired motor coordination, and delays in cognitive and motor development [3]. Specifically, motor skill delays in Down syndrome are characterized by limited motor planning and control, delayed achievement of locomotor milestones, atypical fine motor and writing skills, and reduced manual dexterity [4]. Motor skill delays in childhood, together with physical limitations such as reduced balance, impaired coordination, and lower muscle strength, may hinder participation in daily activities and organized sport [5].

### 1.2. Relevance of Physical Activity

Physical activity is crucial for individuals with Down syndrome, as it supports physical, cognitive, and psychosocial development [6,7,8]. Studies in young adults with Down syndrome have shown that structured physical activity interventions, such as resistance training, can improve motor skills and aspects of cognitive function, even in individuals with mild to moderate impairments [9]. Moreover, regular physical activity positively influences psychological well-being, contributing to improvements in self-esteem, mood, cognitive performance, and overall quality of life [10]. It also promotes social inclusion, peer interaction, development of social skills, and the formation of friendships, while enhancing self-confidence and enjoyment of leisure activities [7]. These benefits are particularly relevant for children and adolescents with Down syndrome, who are at risk of being less active than their typically developing peers [11]. However, social and environmental barriers, including limited access to inclusive programs and societal prejudice, may further restrict opportunities for regular physical activity [5].

### 1.3. Evidence from Intervention Studies

Developing motor skills from an early age is therefore important to promote active participation and prevent functional delays. Sports exercise science professionals play a key role through targeted and progressive interventions aimed at improving fundamental motor skills (FMSs) and supporting the acquisition of essential movement patterns. Motor skills are typically classified into three main categories: locomotor skills, including walking, running, jumping, and climbing or descending steps; non-locomotor skills, such as maintaining balance, bending, and pushing; and object control skills, including throwing, catching, kicking, and manipulating objects [12]. Recent evidence from children with ADHD suggests that different physical activity modalities may have distinct effects on motor, cognitive, and psychosocial outcomes [13]. Personalized interventions, tailored to each child’s abilities, needs, and interests and delivered in inclusive and engaging environments, can improve motor performance, cardiovascular health, coordination, and confidence [5]. In addition, such interventions may promote psychological and social well-being and support cognitive functions, including memory and attention, through self-esteem, motivation, and engagement in group activities [4,14]. In this review, psychosocial outcomes were considered as a broad domain encompassing emotional and behavioural functioning (e.g., aggression, anxiety and depression), attention and social functioning (e.g., attentions problems and social behaviour), and quality of life.

### 1.4. Previous Reviews and Research Gap

Although previous studies have explored the effects of physical activity in individuals with Down syndrome, the available evidence remains fragmented, particularly regarding children and adolescents and the integration of motor and psychosocial outcomes. Previous reviews have largely focused on specific domains, such as balance, physical fitness or motor function, or on specific types of physical activity interventions. Maïano et al. focused specifically on exercise intervention for balance in children and adolescent with Down syndrome [15]. More recently, Lei at al. synthesized eight randomized controlled trials (260 participants) specifically addressing balance in children with Down syndrome and performed a meta-analysis of different exercise modalities [16]. Szabo et al. focused on physical therapy interventions for motor delays in children with Down syndrome, including six heterogeneous studies [17]. Zhang et al. further examined the relationship between exercise dose and multidimensional motor outcomes, including balance, motor skills and coordination, in 23 studies involving children and adolescents with Down syndrome [18]. However, these reviews primarily addressed balance, motor function or physical therapy, whereas a focused synthesis of recent structured physical activity interventions integrating both motor and psychosocial outcomes in children and adolescents with Down syndrome remains limited.

### 1.5. Objective

Therefore, this systematic review aimed to synthesize recent evidence on structured physical activity interventions in children and adolescents with Down syndrome, with a specific focus on motor and psychosocial outcomes. By considering different intervention modalities within the same framework, this review sought to provide an integrated perspective on the potential effects of physical activity across these outcome domains.

## 2. Materials and Methods

### 2.1. Search Strategy

This systematic review was conducted in accordance with the Preferred Reporting Items for Systematic Reviews (PRISMA) 2020 statement (Table S3) [19]. The review protocol was registered in the International Prospective Register of Systematic Reviews (PROSPERO) on 4 September 2025, under the registration number CRD42025114068. A comprehensive literature search was conducted in three electronic databases: PubMed (NLM), Scopus, and Web of Science. The final search was executed on December 15, 2025. Search terms were structured into three thematic groups: Group (A) included the terms “Down syndrome” and “Trisomy 21”. Group B included the terms “physical activity”, “exercise”, “fitness”, “sport”, “aerobic exercise”, and “resistance training”. Group C included the terms “health”, “well-being”, “quality of life”, “physical health”, and “mental health”. Boolean operators “AND” and “OR” were used to combine the search terms across groups. The complete, reproducible search strategy which was adapted for the specific syntax and search fields of each database was: (“Down syndrome” OR “trisomy 21”) AND (“physical activity” OR “exercise” OR “fitness” OR “sport” OR “aerobic exercise” OR “resistance training”) AND (“health” OR “well-being” OR “quality of life” OR “physical health” OR “mental health”). All retrieved articles were imported into EndNote software (version X20 for Windows 11, Thomson Reuters, New York, NY, USA) for duplicate removal and data management.

### 2.2. Inclusion and Exclusion Criteria

Only peer-reviewed full-text articles in English were considered eligible. This language restriction was deliberately applied to ensure accurate interpretation of complex psychosocial, emotional and clinical terminology and to minimize potential semantic ambiguity associated with the use of automated translation tools. Abstracts, conference proceedings, and unpublished materials were excluded. Studies were included if they: (a) involved children and adolescents with Down Syndrome; (b) investigated structured physical activity interventions; (c) reported motor or psychosocial outcomes; (d) were quantitative interventional studies (randomized controlled trials, non-randomized controlled trials, quasi-experimental studies) or observational studies (cohort, cross-sectional); and (e) were published in English within the last 10 years. This 10-year timeframe was defined a priori and specified in the protocol to focus the review on contemporary intervention approaches, methodological standards, and inclusive physical activity frameworks. Although research in this population has a longer history, the selected timeframe was intended to reflect recent developments in bio-psycho-social and inclusive approaches to physical activity. Exclusion criteria included reviews, meta-analyses, editorials, letters, and theses. No restrictions were applied regarding participant gender. The selection of studies was guided by the PICOS framework (Population, Intervention, Comparison, Outcome, and Study design), as summarized in Table 1.

### 2.3. Study Selection

Two independent reviewers screened all identified studies in two phases: (1) title and abstract screening, and (2) full-text assessment for eligibility. Discrepancies between reviewers were resolved through discussion, and when necessary, a third reviewer was consulted to reach consensus. Although formal inter-rater reliability was not calculated, the use of independent screening procedures was implemented to minimize selection bias.

### 2.4. Data Extraction

Data extraction was conducted independently by two reviewers using a standardized spreadsheet. Extracted information included: author and year of publication, sample characteristics (size, age, gender), study objectives, intervention characteristics (type, duration, frequency), and main outcomes. All data were recorded in a Microsoft Excel spreadsheet (Microsoft Corp, Redmond, WA, USA). Any disagreements were resolved through discussion and consensus.

### 2.5. Methodological Quality Assessment

The methodological quality of the included studies was independently assessed by two reviewers using a modified version of the Downs and Black Checklist [20]. This tool was selected because it is applicable to both randomized and non-randomized controlled study designs, making it suitable for the different designs included in this review. In the modified version, item 27, which evaluates statistical power, was simplified from the original 0–5 scale to a binary 0–1 scoring system: a score of 0 was assigned when statistical power was not reported or was below 80%, and a score of 1 when power was ≥ 80%, in accordance with previous methodological recommendations [21]. Consequently, the maximum total score was reduced from 32 to 28. Based on the modified total score, methodological quality was classified as excellent (26–28), good (20–25), fair (15–19), or poor (<15) [22,23]. Disagreements between reviewers were resolved through discussion. In accordance with PRISMA recommendations, detailed item-level judgments are provided in Supplementary Table S1.

### 2.6. Certainty of Evidence

The overall certainty of evidence was assessed using the Grading of Recommendations Assessment, Development and Evaluation (GRADE) framework for each outcome [24]. For GRADE assessment, psychosocial outcomes and quality of life were considered within a single broader psychosocial domain. The initial certainty was determined according to study design, with randomized controlled trials starting at high certainty and non-randomized studies at low certainty. The certainty of evidence was then assessed across five domains: risk of bias, inconsistency, indirectness, imprecision, and publication bias. Evidence was downgraded by one level for serious concerns or by two levels for very serious concerns within each domain. For outcomes supported by a single study, inconsistency was considered not applicable. For psychosocial outcomes, including quality of life, inconsistency was not assessable because the two contributing studies assessed different constructs and did not provide directly comparable outcome measures. The final certainty of evidence was classified as high, moderate, low, or very low. Because quantitative meta-analysis was not feasible, GRADE assessments were based on the available outcome-level evidence from the narrative synthesis and are presented in a Summary of Findings table.

### 2.7. Data Synthesis

Due to the substantial clinical and methodological heterogeneity across interventions and outcome measures, a quantitative synthesis was not considered appropriate. Therefore, findings were synthesized narratively. Studies were grouped according to the main physical activity modality and compared across two primary outcome domains: physical outcomes and psychosocial outcomes. Findings were compared across studies based on the direction and consistency of the reported effects.

## 3. Results

### 3.1. Study Selection

**Figure 1:**
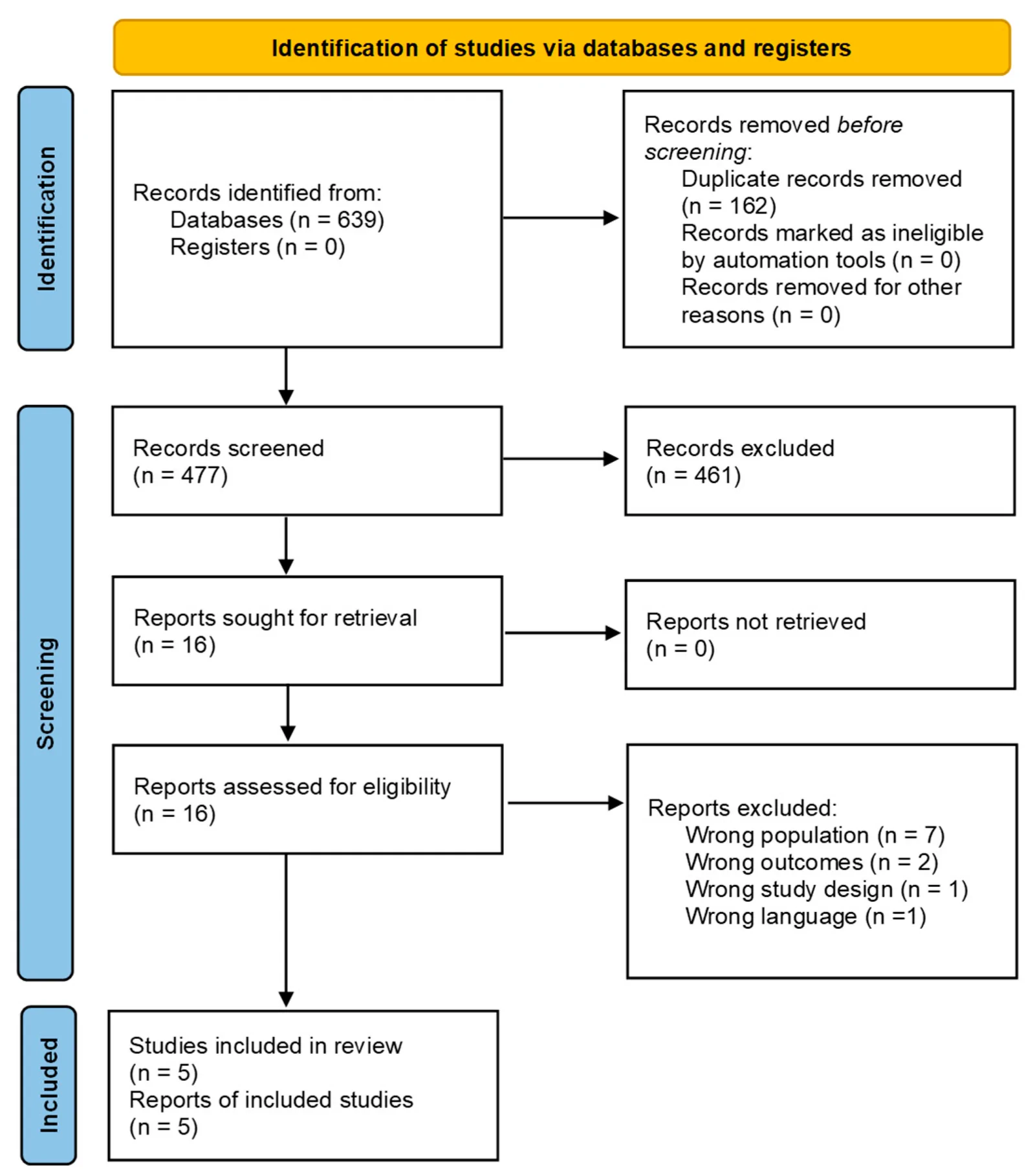
The flow diagram representing the selection process of records.

A total of 639 studies were identified through electronic database searches. After removing 162 duplicates, the titles of 477 studies were screened. Abstracts of 126 articles were then reviewed, resulting in the exclusion of 110 studies. Full texts of the remaining 16 articles were assessed for eligibility, and 5 studies met the predefined inclusion and exclusion criteria. The remaining 11 studies were excluded for specific methodological reasons (e.g., wrong study design, inappropriate population, or focusing on non-eligible outcomes). A detailed list of these 11 excluded full-text articles, along with the primary reason for exclusion for each study, is provided in Supplementary Table S2. The study selection process is summarized in the PRISMA flow diagram (Figure 1).

### 3.2. Study Characteristics

Of the five studies included, one investigated the effects of Pilates exercises [25], one examined core stability and treadmill exercises [26], one focused on training FMSs [27], one evaluated an adapted football program [28], and the last compared traditional Indian dancing with neuromuscular training in children and adolescents with Down syndrome [29]. A total of 186 participants were included across the five studies, of whom 166 had Down syndrome and 20 were typically developing children. Regarding sex distribution, four studies enrolled both male and female participants. Specifically, the reported male-to-female ratios were 12:8 in the Down’s syndrome group and 16:4 in the typically developing group in Capio et al., 21:15 in Raghupathy et al., and 14:26 in Al-Nemr and Reffat. Alsakhawi et al. did not report the exact gender distribution. Perić et al. [28] included only male participants. Importantly, none of the included studies conducted specific sub-analyses to explore potential sex differences in response to the physical activity interventions. Regarding the outcomes, four studies assessed balance, while two studies assessed psychosocial outcomes, including emotional/behavioural variables and quality of life. The methodological quality of the included studies was assessed using a modified version of the Downs and Black checklist. Of the five studies, three were rated as “fair quality” [25,26,27], while two were classified as “good quality” [28,29]. Further details of the included studies are presented in Table 2.

### 3.3. Certainty of Evidence

The certainty of evidence was assessed using the GRADE approach across five domains: risk of bias, inconsistency, indirectness, imprecision and publication bias. Overall, the certainty of evidence was rated as low for balance/postural control, motor skills/fundamental movement skills and psychosocial outcomes (Table 3). Risk of bias was downgraded by one level because several studies had limitations in the reporting or implementation of randomization, allocation concealment, and blinding procedures. Inconsistency was not downgraded for outcomes supported by multiple studies. For psychosocial outcomes, including quality of life, inconsistency was not assessable because the two contributing studies assessed different constructs and did not provide directly comparable outcome measures. Indirectness and imprecision were each downgraded by one level because of differences in participant characteristics, intervention protocols, and outcome measures, as well as the small number and sample size of the included studies. Publication bias could not be formally assessed because of the limited number of studies.

### 3.4. Pilates Exercise

Among the identified studies, only one applied this method to children with Down syndrome. Al-Nemr and Reffat [25] conducted a study with 40 children, randomly assigned to a control group (*n* = 20) or an experimental group (*n* = 20). Both groups participated in a physical therapy program, with the experimental group receiving an additional 45-min Pilates component. Both groups trained three times per week for 12 weeks. The Pilates exercises targeted the strengthening of trunk and lower limb stabilizing muscles, with particular attention to spinal and pelvic alignment and breathing rhythm. Between-group comparisons at post-intervention showed significantly greater improvements in the Pilates group across measures of dynamic balance, gross motor coordination, and quality of life. These findings suggest that adding Pilates to a conventional physical therapy program may provide additional benefits across motor and quality of life outcomes [30].

### 3.5. Core Stability and Treadmill Training

Among the included studies, only Alsakhawi and Elshafey [26] investigated the effectiveness of core muscle training for improving balance and postural stability in children with Down syndrome. This randomized controlled trial included 45 children with Down syndrome, divided into three groups of 15 participants each. Group A received a 60-min traditional physical therapy program targeting postural control and balance; Group B received 30 min of the traditional physical therapy plus 30 min of core stability exercise; and Group C received 30 min of the traditional physical therapy plus 30 min of treadmill training. All groups trained three times per week for 8 weeks. Functional balance and postural stability improved significantly within all three groups. Post-intervention comparisons showed greater improvements in Groups B and C than in Group A, whereas no significant differences were found in Groups B and C.

### 3.6. Fundamental Motor Skills Training

Capio et al. [27] examined the relationship between FMS proficiency and balance in children with Down syndrome, comparing them with children with typical development. The study also evaluated the effects of an FMS training program aimed at improving specific motor skills on both FMS performance and balance. A total of 40 children participated, including 20 with Down syndrome and 20 typically developing children. The training program was based on the principle of error reduction and consisted of two 45-min sessions per week over five weeks (10 sessions in total). Each week included one session focused on locomotor skills and one on object control skills. Activities were conducted in small groups of 3–5 children, organized into circuits with four stations targeting specific skills. The intervention group showed significant pre-to post-intervention improvements in FMS proficiency, including both locomotor and object-control skills, as well as selected measures of postural balance. However, because the study did not include a control training group, these within-group changes cannot be interpreted as evidence of a between-group intervention effect.

### 3.7. Adapted Soccer Program

Perić et al. [28] investigated the effects of an adapted soccer program on motor learning and selected psychosocial characteristics in adolescents with Down syndrome. Participants were randomly assigned to an exercise group (*n* = 12) or a control group (*n* = 13). The program lasted 16 weeks. During the study period, the control group maintained their usual daily activities without additional exercise, while the exercise group participated in two weekly sessions of adapted soccer, each consisting of a 10-min warm-up, 45 min of specific training, and a 5-min cool-down. Psychosocial and motor variables were assessed before and after the intervention. Between-group analyses showed significant time-by-group effects for aggression, attention disorders, anxiety/depression, and social problems, favouring the adapted soccer group. For motor outcomes, a significant improvement in time-by-group effect was observed only for the straight-dribbling task, whereas the other motor tasks did not show significant between-group effects. These results suggest that the adapted soccer program may have greater effects on psychosocial outcomes than on motor performance in adolescents with Down syndrome.

### 3.8. Dancing vs. Neuromuscular Training

Raghupathy et al. [29] compared the effects of traditional dance with a neuromuscular training program on motor skills and balance in children with Down syndrome. In this randomized controlled trial, participants were randomly assigned to an experimental group, which followed a structured dance program (*n* = 18), or a control group, which followed a neuromuscular training program (*n* = 18). The dance group received a structured traditional Indian dance program, while the control group participated in neuromuscular training. The neuromuscular training group performed physical activity interventions targeting balance, muscle strengthening, and coordination. Both groups completed three 60-min sessions per week (including 10-min warm-up and cool-down) over six weeks. Between-group analyses showed significantly greater improvements in gross motor quotient, locomotor skills, and Four-Square Step Test performance in the dance group, with large effect sizes (Cohen’s d = 2.1 for GM1, 2.1 for locomotor skills, and 2.0 for the Four-Square Step Test). The between-group effect for object control was moderate (d = 0.7), whereas no meaningful between-group difference was observed for the Paediatric Balance Scale (d = 0.1).

## 4. Discussion

This systematic review aimed to examine the effects of physical activity in children and adolescents with Down syndrome, focusing on recent evidence across both motor and psychosocial outcomes. Recent reviews have examined physical activity in this population, often focusing on specific outcomes such as balance, motor function, or physical fitness. In contrast, the present review considered different physical activity modalities examining motor and psychosocial outcomes. However, only five studies met our eligibility criteria, highlighting the limited and heterogeneous evidence currently available. These findings should be interpreted in the context of previous systematic reviews. Maïano et al. included 11 studies and focused specifically on exercise interventions targeting balance in children and adolescents with Down syndrome, reporting benefits mainly for static and static–dynamic balance [15]. Lei et al. reported significant improvements in dynamic and overall balance following different exercise modalities, including treadmill, core stability, Pilates, and isokinetic interventions [16]. Szabo et al. focused on physical therapy interventions and suggested greater effectiveness during the early stages of motor development in children with Down Syndrome [17]. In comparison, the present review considered a broader range of structured physical activity modalities, including Pilates, core and treadmill training, FMS training, adapted football, and traditional dance, and found that these interventions may be associated with improvements in both motor and psychosocial outcomes. However, the interventions varied considerably in their objectives, characteristics, and outcome measures, limiting direct comparisons across studies. Motor-oriented interventions, such as Pilates, core stability training, treadmill-based exercise, and FMS programs, primarily targeted physical domains, including balance, postural control, coordination, and motor competence. In contrast, the adapted football intervention assessed both motor and psychosocial outcomes and showed pronounced effects on psychosocial variables.

However, it is crucial to emphasize that while the evidence for motor improvements is supported by multiple studies, the evidence for psychosocial outcomes, including quality of life, remains limited and is based on only two studies assessing different constructs. Therefore, conclusions regarding psychosocial and quality of life outcomes should be interpreted with caution.

Consistent with the previous literature, structured physical activity appears to improve coordination, muscle strength, and locomotor skills in children and adolescents with Down Syndrome [15,16,31]. In particular, multiple interventions were associated with improvements in balance and postural stability. For instance, core stability exercises and treadmill training were associated with improvements in functional balance, while traditional Indican dance produced balance improvements comparable to neuromuscular training [25,26,29]. These factors are relevant given the balance impairments commonly observed in children with Down syndrome, which may be related to hypotonia, ligament laxity, and structural differences [32,33]. Regarding motor skill acquisition, Capio et al. [27] highlighted a relationship between FMS proficiency and balance in children with Down syndrome. Although the authors discussed a possible role of working memory in motor learning, this mechanism was not directly tested and should therefore be considered hypothesis-generating. Perić et al. [28] investigated adapted football and found more pronounced changes in psychosocial outcomes, such as reduced anxiety, aggression, and depressive symptoms, than for motor skills. The group-based nature of adapted football may partly contribute to the observed psychosocial effects, although this mechanism was not directly assessed [34,35]. The limited improvements in motor outcomes may reflect, among other factors, the tasks-specific demands of football training; however, this interpretation remains speculative. This underlines the importance of balancing motor and psychosocial objectives when designing these programs [36]. Finally, Raghupathy et al. [29] found that traditional Indian dance was associated with greater improvements in gross motor and locomotor skills than neuromuscular training, whereas balance outcomes were comparable between the two interventions. It is important to note that the included interventions varied substantially in type, duration, intensity, and outcome measures. This heterogeneity limited direct comparability across studies and precluded meaningful identification of a single most effective intervention. Therefore, a quantitative synthesis was not considered appropriate, and the findings were synthesized narratively. A more in-depth critical appraisal of the included studies highlights several methodological limitations. Most studies had small sample sizes, reducing statistical power. In addition, key methodological elements such as randomization procedures, allocation concealment, and blinding were often insufficiently reported, introducing potential risk of bias. Intervention protocols and outcome measures were also highly heterogeneous, further limiting comparability across studies and strengthening the decision not to perform a meta-analysis. Consistent with the GRADE assessment, the overall certainty of evidence was low, supporting a cautious interpretation of the observed effects.

Although the wide age range (4–17 years) increases heterogeneity in terms of developmental and psychosocial characteristics, and the small number of studies did not allow age-specific subgroup analyses, some age-related differences emerged from the narrative synthesis. Studies involving younger children (4–10 years) mainly focused on fundamental motor skills, balance, and postural control through individual or small-group activities (e.g., Pilates, treadmill training, and dance). In contrast, the study including adolescents (15–17 years) focused on team sports (e.g., adapted football) and mainly addressed psychosocial outcomes, such as peer interaction and emotional regulation. These patterns may indicate that intervention goals could be tailored to developmental and social characteristics across age groups; however, this hypothesis should be investigated in larger studies. Recent longitudinal evidence from a general adolescent population also suggests a potential link between physical activity, body image, and social anxiety. However, these findings derive from adolescents without Down syndrome and therefore provide contextual rather than population-specific evidence [37].

### 4.1. Strengths and Limitations

This review provides a focused synthesis of recent physical activity interventions targeting both motor and psychosocial outcomes in children and adolescents with Down syndrome. A major strength lies in the inclusion of diverse intervention types, allowing for a broader understanding of how different physical activity modalities may influence development. In addition, both motor and psychosocial outcomes were considered, offering a more holistic perspective compared to previous reviews focused on single domains.

Several limitations should be acknowledged. First, the included studies covered a broad age range (4–17 years) encompassing participants at substantially different neurodevelopmental and psychosocial stages. Therefore, the observed effects may not be consistent across age groups and should be interpreted as an overall indication of the potential benefits of physical activity rather than as age-specific developmental effects. The limited number of available studies prevented meaningful age-based subgroup analysis. Second, the inclusion of terms related to health, well-being, or quality of life in the search strategy may have limited the retrieval of studies focused primarily on specific motor or functional outcomes, such as gait, balance, postural control or motor development. Although broad physical activity terms captured diverse exercise modalities, including Pilates, dance and treadmill training, studies addressing isolated motor or biomechanical domains may have been underrepresented. Future reviews focusing specifically on motor and functional outcomes should consider more comprehensive and outcome-specific search strategies to facilitate the identification of relevant studies. Third, the a priori decision to restrict the search to the last 10 years may have excluded relevant earlier studies. Thus, the findings should be interpreted as a synthesis of recent evidence rather than an exhaustive overview of all physical activity interventions in this population.

The small number of included studies (*n* = 5) further limits the robustness and generalizability of the findings. In addition, the methodological quality of the included studies was generally moderate, indicating a potential risk of bias. Heterogeneity in intervention characteristics and outcome measures further limits the ability to draw firm conclusions regarding the relative effectiveness of specific approaches. Evidence regarding psychosocial outcomes was particularly limited, as only two studies assessed these outcomes, precluding broader conclusions in this domain. A further limitation is that some studies identified in more recent reviews were not retrieved by our search strategy, which may have resulted in an incomplete identification of eligible evidence. Finally, although study selection and quality appraisal were conducted independently by the reviewers, formal inter-rater reliability was not calculated. Given the small number of studies ultimately included, this may represent an additional methodological limitation and should be considered when interpreting the robustness of the findings.

### 4.2. Future Directions

Based on the current findings and identified limitations, future research should focus on high-quality randomized controlled trials with larger sample sizes and longer follow-up periods to strengthen the available evidence. Standardization of intervention protocols and outcome measures would improve comparability across studies and facilitate quantitative synthesis. Given the limited evidence identified in this review, particular attention should be given to psychosocial outcomes and quality of life, which were assessed in only a small proportion of the included studies. Future studies should also report sex-disaggregated outcomes, as none of the included studies examined potential differences in responses between male and female participants. More consistent reporting of intervention characteristics and follow-up data would further support the development of robust evidence-based recommendations for physical activity in children and adolescents with Down syndrome.

## 5. Conclusions

In conclusion, structured physical activity interventions may be associated with improvements in motor outcomes, while preliminary evidence suggests potential benefits for psychosocial outcomes in children and adolescents with Down Syndrome. However, these findings are based on only two studies assessing different constructs and therefore remain uncertain. These findings should be interpreted with caution due to the limited number of available studies, methodological variability, and heterogeneity in interventions and outcomes. Overall, the certainty of evidence was low, indicating limited confidence in the observed effects. Further high-quality studies with larger samples and standardized outcome measures are needed to clarify the effects of physical activity and strengthen the evidence base for clinical practice.

## Figures and Tables

**Table 1:** The PICOS framework.

PICOS Component	Details
Population	Children and adolescents with Down syndrome
Intervention	Physical activity intervention
Comparator	Outcomes in the intervention group compared with those in the control group, alternative physical activity interventions, typically developing peers, or not applicable (for descriptive/observational studies)
Outcome	Motor development (including balance and fundamental motor skills) and psychosocial outcomes (including quality of life, emotional regulation and social behaviour), regardless of the direction of the reported effects.
Study Design	Included: Quantitative interventional studies (randomized controlled trials, non-randomized controlled trials, quasi-experimental studies) and observational studies (cohort, cross-sectional). Excluded: Reviews, meta-analyses, qualitative studies, case reports, editorials, and letters.

**Table 2:** The aim of the studies and relative characteristics of the samples.

Study, Country & Setting	Study Design	Participants	Intervention	Comparator	Outcomes & Instruments	Follow-Up Time Points	Main Results	D&B Score
Capio et al. [27], 2018; Hong Kong; DS advocacy group/community setting	Two-part study: (1) cross-sectional comparison between children with DS and TD children; (2) single-group pre–post intervention study in children with DS. No control training was implemented	N = 40: DS, *n* = 20; TD, *n* = 20. DS children were aged 7.10 ± 2.90.	5 weeks; 2 sessions/week; 45 min/session; 10 sessions. Circuit-based FMS training targeting locomotor and object-control skills using an error-reduced approach.	No training control group for the intervention phase. TD participants were used only for the initial cross-sectional comparison.	FMS: TGMD-2. Balance: force platform/COP parameters. Short-term memory: forward digit recall and Corsi block tapping tests.	Baseline and immediately after the 5-week intervention.	Overall FMS: F = 54.01, *p* < 0.001, η^2^ = 0.87. Locomotor: F = 13.57, *p* = 0.002, η^2^ = 0.44. Object control: F = 114.31, *p* < 0.001, η^2^ = 0.87. Balance improved for COP AV (*p* = 0.027), PL (*p* = 0.026), and SD-AP (*p* = 0.045), but not SD-ML (*p* = 0.954).	17
Alsakhawi & Elshafey [26], 2019; Egypt; outpatient clinic, Faculty of Physical Therapy	Randomized controlled trial with three parallel groups.	N = 45, children with DS aged 4–6 years; *n* = 15/group.	Group A: traditional PT and balance exercises. Group B: traditional PT + core stability training. Group C: traditional PT + treadmill training	Group A: traditional PT/balance programme. Group B: traditional PT + core stability. Group C: traditional PT + treadmill.	Functional balance: BBS. Postural stability: Biodex Balance System Overall Stability Index.	Pre-treatment and immediately post-treatment.	Functional balance: Group A 32.5 ± 3.8 → 38.0 ± 2.58; Group B 31.7 ± 3.3 → 43.8 ± 2.91; Group C 33.2 ± 2.5 → 45.0 ± 2.12; all within-group *p* = 0.001. Overall Stability Index: A 4.20 ± 0.40 → 5.40 ± 0.70; B 4.60 ± 0.289 → 6.90 ± 0.33; C 5.10 ± 0.15 → 7.40 ± 0.54; all *p* = 0.001. No significant difference between Groups B and C after treatment.	18
Perić et al. [28], 2022; Serbia; Creative Educative Centre	Randomized two-group controlled pre–post study.	N = 25, all male adolescents with DS aged 15–17 years. Exercise group: *n* = 12; control: *n* = 13. Mild ID: 75% vs. 77%; moderate ID: 25% vs. 23%	Adapted soccer; 16 weeks; 2 sessions/week; 60 min/session. Supervised soccer-specific motor-learning programme.	Usual daily regime with no additional systemic exercise.	Psychosocial characteristics: 51-item observation sheet assessing aggression, attention disorders, anxiety/depression, and social problems. Motor learning: straight dribbling, dribbling with ball stopping, and slalom with goal kicking.	Pre-intervention and immediately post-intervention.	Significant time–group interactions for aggression (F = 17.93, *p* < 0.001, η^2^*p* = 0.499), attention (F = 17.52, *p* = 0.001, η^2^*p* = 0.493), anxiety/depression (F = 16.76, *p* = 0.001, η^2^*p* = 0.482) and social problems (F = 12.80, *p* = 0.002, η^2^*p* = 0.416). Straight dribbling improved from 1.55 ± 1.04 to 2.45 ± 1.13 (F = 59.80, *p* < 0.001, η^2^p = 0.722).	20
Raghupathy et al. [29], 2022; India; Special schools	Randomized, double-arm controlled trial.	N = 36, children with DS aged 6–10 years; Indian dance *n* = 18; neuromuscular training *n* = 18. Dance: 11 boys/7 girls; control: 10 boys/8 girls.	Traditional Indian dance; 6 weeks; 3 sessions/week; 60 min/session, including 10-min warm-up/cool-down.	Neuromuscular training with balance, strengthening, coordination, walking/hopping/kicking, obstacle-course and ball-manipulation exercises.	Gross motor skills: TGMD-2 (GMQ, locomotor, object control). Dynamic balance: FSST. Functional balance: PBS.	Baseline and 6 weeks post-training.	Significant time–group interaction for TGMD-2 GMQ (F = 38.9, *p* < 0.001, η^2^ = 0.549), locomotor (F = 37.8, *p* < 0.001, η^2^ = 0.541) and FSST (F = 34.9, *p* < 0.001, η^2^ = 0.521). Change scores: GMQ 30.47 vs. 11.10; locomotor 11.10 vs. 4.35; FSST 4.29 vs. 2.41, favouring dance. Object control: *p* = 0.068. PBS: *p* = 0.692. Cohen’s d: 2.1 for GMQ, 2.1 for locomotor, 2.0 for FSST, 0.7 for object control and 0.1 for PBS.	22
Al-Nemr & Reffat [25], 2024; Egypt; outpatient clinic, Faculty of Physical Therapy	Single-blinded randomized controlled trial.	N = 40, children with DS aged 8–10 years; Pilates *n* = 20; control *n* = 20. Pilates: 8 boys/12 girls; control: 6 boys/14 girls. Mean age: 8.86 ± 0.58 vs. 9.16 ± 0.48 years.	12 weeks; 3 sessions/week; 90 min/session total. Both groups received designed PT for 45 min; Pilates group received an additional 45-min Pilates component.	Designed PT programme, including flexibility, strengthening, walking and postural-control exercises	Dynamic balance: Biodex Balance System. Gross motor coordination: BOT-2. Quality of life: PedsQL Generic Core Scale, parent-proxy report.	Baseline and immediately post-treatment.	Significant treatment–time interaction: F(9,30) = 116.3, *p* < 0.0001, η^2^ = 0.972. Post-treatment differences favoured Pilates for all stability indices (all *p* < 0.0001), BOT-2 body coordination (*p* < 0.0001), strength/agility (*p* = 0.03), gross motor composite (*p* = 0.018), physical functioning (*p* = 0.001), psychosocial (*p* < 0.0001) and total PedsQL (*p* < 0.0001).	19

Notes: (DS) Down syndrome; (TD) typically developing; (N) total sample size; (n) subgroup sample size; (PT) physical therapy; (FMSs) fundamental movement skills; (TGMD-2) Test of Gross Motor Development-Second Edition; (GMQ) Gross Motor Quotient; (COP) center of pressure; (AV) average velocity; (PL) path length; (SD-ML) standard deviation of medial–lateral COP displacement; (SD-AP) standard deviation of anterior–posterior COP displacement; (BBS) Berg Balance Scale; (FSST) Four-Square Step Test; (PBS) Pediatric Balance Scale; (BOT-2) Bruininks–Oseretsky Test of Motor Proficiency, Second Edition; (PedsQL) Pediatric Quality of Life Inventory; (ID) intellectual disability; (η^2^) eta squared; (η^2^*p*) partial eta squared; (d) Cohen’s d.

**Table 3:** Summary of findings and certainty of evidence according to the GRADE approach.

Outcome	No. of Studies (Participants)	Risk of Bias	Inconsistency	Indirectness	Imprecision	Publication Bias	Overall Certainty
Balance/postural control	4 (141)	Serious (−1)	Not serious	Serious (−1)	Serious −1	Not assessable	◯⊕◯◯ Low
Motor Skills/Fundamental movement skills	4 (121)	Serious (−1)	Not serious	Serious (−1)	Serious −1	Not assessable	◯⊕◯◯ Low
Psychosocial Outcomes	2 (65)	Serious (−1)	Not assessable	Serious (−1)	Serious (−1)	Not assessable	◯⊕◯◯ Low

Note. Initial certainty was high for randomized controlled trials and low for non-randomized evidence. Publication bias was not formally assessable because of the limited number of included studies. Psychosocial outcomes included emotional and behavioural functioning, attention and social functioning, and quality of life. Inconsistency was not assessable because the two contributing studies assessed different psychosocial constructs and did not provide directly comparable outcome measures.

## Data Availability

No new data were created or analyzed in this study. Data sharing is not applicable to this article.

## Supplementary Materials

The following supporting information can be downloaded at: https://www.mdpi.com/article/10.3390/jcm15176823/s1, Table S1: Quality assessment of the included studies; Table S2: Excluded full-text articles and primary reasons for exclusion; Table S3: PRISMA 2020 Checklist.

## References

[1] Blanco-Montaño A., Ramos-Arenas M., Yerena-Echevarría B.A., Miranda-Santizo L.D., Ríos-Celis A.L., Dorantes-Gómez A.T., Morato-Rangel A.J., Meza-Hernández J.A., Acosta-Saldívar E.D., Aguilar-Castillo C.D. (2023). Risk factors in the origin of Down syndrome. Rev. Med. Inst. Mex. Seguro Soc..

[2] Antonarakis S.E., Skotko B.G., Rafii M.S., Strydom A., Pape S.E., Bianchi D.W., Sherman S.L., Reeves R.H. (2020). Down syndrome. Nat. Rev. Dis. Primers.

[3] Wajuihian S. (2016). Down syndrome: An overview. Afr. Vis. Eye Health.

[4] Alesi M., Battaglia G., Lanfranchi S. (2019). Chapter Six—Motor development and Down syndrome. International Review of Research in Developmental Disabilities.

[5] Barr M., Shields N. (2011). Identifying the barriers and facilitators to participation in physical activity for children with Down syndrome. J. Intellect. Disabil. Res..

[6] Paul Y., Ellapen T.J., Barnard M., Hammill H.V., Swanepoel M. (2019). The health benefits of exercise therapy for patients with Down syndrome: A systematic review. Afr. J. Disabil..

[7] Muñoz-Llerena A., Ladrón-de-Guevara L., Medina-Rebollo D., Alcaraz-Rodríguez V. (2024). Impact of Physical Activity on Autonomy and Quality of Life in Individuals with Down Syndrome: A Systematic Review. Healthcare.

[8] Andriolo R.B., El Dib R.P., Ramos L., Atallah A.N., da Silva E.M. (2010). Aerobic exercise training programmes for improving physical and psychosocial health in adults with Down syndrome. Cochrane Database Syst. Rev..

[9] Post E.M., Kraemer W.J., Kackley M.L., Caldwell L.K., Volek J.S., Sanchez B.N., Focht B.C., Newton R.U., Häkkinen K., Maresh C.M. (2022). The Effects of Resistance Training on Physical Fitness and Neuromotor-Cognitive Functions in Adults with Down Syndrome. Front. Rehabil. Sci..

[10] Mahindru A., Patil P., Agrawal V. (2023). Role of Physical Activity on Mental Health and Well-Being: A Review. Cureus.

[11] Fox B., Moffett G.E., Kinnison C., Brooks G., Case L.E. (2019). Physical Activity Levels of Children With Down Syndrome. Pediatr. Phys. Ther..

[12] Wentz E.E., Looper J., Menear K.S., Rohadia D., Shields N. (2021). Promoting Participation in Physical Activity in Children and Adolescents with Down Syndrome. Phys. Ther..

[13] Li D., Wang D., Cui W., Yan J., Zang W., Li C. (2023). Effects of different physical activity interventions on children with attention-deficit/hyperactivity disorder: A network meta-analysis of randomized controlled trials. Front. Neurosci..

[14] Catama B. (2024). Promoting Physical Activity for Children with Down Syndrome: An Evidence-Based Framework. https://papers.ssrn.com/sol3/papers.cfm?abstract_id=4899540.

[15] Maïano C., Hue O., Lepage G., Morin A.J.S., Tracey D., Moullec G. (2019). Do Exercise Interventions Improve Balance for Children and Adolescents with Down Syndrome? A Systematic Review. Phys. Ther..

[16] Lei Z., Yuan K., Xu J., Miao Y., Dai Y., Wang J., Chang J. (2025). Effects of physical exercises on balance in children with down syndrome: A systematic review and meta-analysis. BMC Sports Sci. Med. Rehabil..

[17] Szabo D.A., Stoian A., Veres C., Adumitrachioaie H., Pârvu C., Hășmășan I.T., Sopa I.S. (2026). The Effects of Physical Therapy in the Rehabilitation of Motor Delays in Children with Down Syndrome: A Systematic Review. J. Clin. Med..

[18] Zhang X., Xiong Q., Weng S. (2026). Exercise dose and multidimensional motor function in young people with Down syndrome: A Bayesian network dose–response meta-analysis. Pediatr. Res..

[19] Page M.J., McKenzie J.E., Bossuyt P.M., Boutron I., Hoffmann T.C., Mulrow C.D., Shamseer L., Tetzlaff J.M., Akl E.A., Brennan S.E. (2021). The PRISMA 2020 statement: An updated guideline for reporting systematic reviews. BMJ.

[20] Downs S.H., Black N. (1998). The feasibility of creating a checklist for the assessment of the methodological quality both of randomised and non-randomised studies of health care interventions. J. Epidemiol. Community Health.

[21] Chuter V., Spink M., Searle A., Ho A. (2014). The effectiveness of shoe insoles for the prevention and treatment of low back pain: A systematic review and meta-analysis of randomised controlled trials. BMC Musculoskelet. Disord..

[22] Pas H.I., Reurink G., Tol J.L., Weir A., Winters M., Moen M.H. (2015). Efficacy of rehabilitation (lengthening) exercises, platelet-rich plasma injections, and other conservative interventions in acute hamstring injuries: An updated systematic review and meta-analysis. Br. J. Sports Med..

[23] Hooper P., Jutai J.W., Strong G., Russell-Minda E. (2008). Age-related macular degeneration and low-vision rehabilitation: A systematic review. Can. J. Ophthalmol..

[24] Guyatt G.H., Oxman A.D., Vist G.E., Kunz R., Falck-Ytter Y., Alonso-Coello P., Schünemann H.J. (2008). GRADE: An emerging consensus on rating quality of evidence and strength of recommendations. BMJ.

[25] Al-Nemr A., Reffat S. (2024). Effect of Pilates exercises on balance and gross motor coordination in children with Down syndrome. Acta Neurol. Belg..

[26] Alsakhawi R.S., Elshafey M.A. (2019). Effect of Core Stability Exercises and Treadmill Training on Balance in Children with Down Syndrome: Randomized Controlled Trial. Adv. Ther..

[27] Capio C.M., Mak T.C.T., Tse M.A., Masters R.S.W. (2018). Fundamental movement skills and balance of children with Down syndrome. J. Intellect. Disabil. Res..

[28] Perić D.B., Milićević-Marinković B., Djurović D. (2022). The effect of the adapted soccer programme on motor learning and psychosocial behaviour in adolescents with Down syndrome. J. Intellect. Disabil. Res..

[29] Raghupathy M.K., Divya M., Karthikbabu S. (2022). Effects of Traditional Indian Dance on Motor Skills and Balance in Children with Down syndrome. J. Mot. Behav..

[30] Lee K. (2025). Effects of Core Stability Training on Deep Stabilizing Muscle Function and Neuromuscular Control. Medicina.

[31] Melo G.L.R., Neto I.V.S., da Fonseca E.F., Stone W., Nascimento D.D.C. (2022). Resistance training and Down Syndrome: A narrative review on considerations for exercise prescription and safety. Front. Physiol..

[32] Jain P.D., Nayak A., Karnad S.D., Doctor K.N. (2022). Gross motor dysfunction and balance impairments in children and adolescents with Down syndrome: A systematic review. Clin. Exp. Pediatr..

[33] Jain P.D., Nayak A., Karnad S.D. (2022). Relationship between trunk muscle strength, reaching ability and balance in children with Down syndrome—A cross-sectional study. Brain Dev..

[34] Garaigordobil M., Berrueco L., Celume M.P. (2022). Developing Children’s Creativity and Social-Emotional Competencies through Play: Summary of Twenty Years of Findings of the Evidence-Based Interventions “Game Program”. J. Intell..

[35] Zhao Z., Che X., Wang H., Zheng Y., Ma N., Gao L., Shui Y. (2025). The Relationship Between Soccer Participation and Team Cohesion for Adolescents: A Chain-Mediated Effect of Athlete Engagement and Collective Self-Esteem. Behav. Sci..

[36] Rutter T.L., Hastings R.P., Murray C.A., Enoch N., Johnson S., Stinton C. (2024). Psychological wellbeing in parents of children with Down syndrome: A systematic review and meta-analysis. Clin. Psychol. Rev..

[37] Xing S., Yang Y., Wu J., Shao Y., Zang W. (2026). Can physical exercise alleviate social anxiety in junior high school students by improving body image?. Front. Psychol..

