# Supplementary material for: Effects of Physical Activity on Motor Development, Balance, and Psychosocial Well-Being in Children and Adolescents with Down Syndrome: A Systematic Review
Source: J Clin Med. 2026 Sep 3;15(17):6823. doi: 10.3390/jcm15176823 (PMC13565851; doi:10.3390/jcm15176823)
Supplement: Supplementary file 1 [file jcm-15-06823-s001.zip › jcm-4498974-supplementary.pdf]

Supplementary Table S1. Quality assessment of the included studies.

**First Author (year)**	1	2	3	4	5	6	7	8	9	10	11	12	13	14	15	16	17	18	19	20	21	22	23	24	25	26	27	**Total Score**
**Capio et al. (2018)**	1	1	1	1	2	1	1	0	1	1	0	0	1	0	1	1	1	1	0	1	0	0	0	0	1	0	0	17
**Alsakhawi et al. (2019)**	1	1	1	1	2	1	1	0	1	0	0	0	1	0	0	1	1	1	0	1	1	1	1	0	1	0	0	18
**Peric et al. (2022)**	1	1	1	1	2	1	1	1	1	1	0	0	1	0	0	1	1	1	1	0	1	1	1	0	1	1	0	20
**Raghupathy et al. (2022)**	1	1	1	1	2	1	1	1	0	1	0	0	0	0	1	1	1	1	1	1	1	1	1	0	1	1	1	22
**Al-Nemr et al. (2024)**	1	1	1	1	2	1	1	0	0	1	0	0	0	0	1	1	1	1	0	1	0	1	1	1	1	0	1	19

U, unable to determine; 1, yes; 0, no. For item 5: 0, no; 1, partially; 2, yes.

Downs and Black checklist items: *Reporting [(1) Is the hypothesis/aim/objective of the study clearly described?; (2) Are the main outcomes to be measured clearly described in the Introduction or Methods section?; (3) Are the characteristics of the patients included in the study clearly described?; (4) Are the interventions of interest clearly described?; (5) Are the distributions of principal confounders in each group of subjects to be compared clearly described?; (6) Are the main findings of the study clearly described?; (7) Does the study provide estimates of the random variability in the data for the main outcomes?; (8) Have all important adverse events that may be a consequence of the intervention been reported?; (9) Have the characteristics of patients lost to follow-up been described?; (10) Have actual probability values been reported (e.g., 0.035 rather than <0.05) for the main outcomes except where the probability value is less than 0.001?]; External validity [(11) Were the subjects asked to participate in the study representative of the entire population from which they were recruited?; (12) Were those subjects who were prepared to participate representative of the entire population from which they were recruited?; (13) Were the staff, places, and facilities where the patients were treated, representative of the treatment the majority of patients receive?]; Internal validity - bias [(14) Was an attempt made to blind study subjects to the intervention they have received?; (15) Was an attempt made to blind those measuring the main outcomes of the intervention?; (16) If any of the results of the study were based on "data dredging," was this made clear?; (17) In trials and cohort studies, do the analyses adjust for different lengths of follow-up of patients, or in case-control studies, is the time period between the intervention and outcome the same for cases and controls?; (18) Were the statistical tests used to assess the main outcomes appropriate?; (19) Was compliance with the intervention/s reliable?; (20) Were the main outcome measures used accurate (valid and reliable)?]; Internal validity - confounding (selection bias) [(21) Were the patients in different intervention groups (trials and cohort studies) or were the cases and controls (case-control studies) recruited from the same population?; (22) Were study subjects in different intervention groups (trials and cohort studies) or were the cases and controls (case-control studies) recruited over the same period of time?; (23) Were study subjects randomized to intervention groups?; (24) Was the randomized intervention assignment concealed from both patients and health care staff until recruitment was complete and irrevocable?; (25) Was there adequate adjustment for confounding in the analyses from which the main findings were drawn?; (26) Were losses of patients to follow-up taken into account?]; (27) Power: Did the study have sufficient power to detect a clinically important effect where the probability value for a difference being due to chance is less than 5%*

Supplementary Table S2. Excluded full-text articles and primary reasons for exclusion.

Excluded Article (Full Reference)	Primary Reason for Exclusion
Alesi, M. and G. Battaglia (2019). Motor development and Down syndrome. International Review of Research in Developmental Disabilities, Vol 56: State of the Art of Research on down Syndrome. S. Lanfranchi. 56: 169-211.	Wrong study design: Book chapter / Narrative Review
Alesi, M., et al. (2022). "Motor Coordination and Global Development in Subjects with Down Syndrome: The Influence of Physical Activity." Journal of Clinical Medicine 11(17).	Wrong population: Study conducted on adults.
Boer, P. H. and Z. de Beer (2019). "The effect of aquatic exercises on the physical and functional fitness of adults with Down syndrome: A non-randomised controlled trial." J Intellect Disabil Res 63(12): 1453-1463.	Wrong population: Study conducted on adults.
*Campagna, J., et al. (2024). "*The physical and psychosocial impact of a school-based running programme for adolescents with disabilities." Journal of Intellectual Disability Research 68(2): 181-192.	Wrong population: Mixed disabilities cohort.
Chen, C. C. and S. D. R. Ringenbach (2016). "Dose-response relationship between intensity of exercise and cognitive performance in individuals with Down syndrome: A preliminary study." Journal of Intellectual Disability Research 60(6): 606-614.	Wrong outcome: Cognitive focus.
Kashi, A., et al. (2023). "The Effect of an Exercise Package for Students with Intellectual Disability on Motor and Social Development." Iranian Journal of Child Neurology 17(2): 93-110.	Wrong population: General Intellectual Disability.
Merzbach, V., et al. (2023). "Impact of Prescribed Exercise on the Physical and Cognitive Health of Adults with Down Syndrome: The MinDSets Study." International Journal of Environmental Research and Public Health 20(23)	Wrong population: Study conducted on adults.
Pando, E. V. B., et al. (2023). "Physical activity in people with Down Syndrome." Retos-Nuevas Tendencias En Educacion Fisica Deporte Y Recreacion(50): 415-420.	Wrong language: Full text published in Spanish.
Park, S. B., et al. (2022). "Effect of a Cognitive Function and Social Skills-Based Digital Exercise Therapy Using IoT on Motor Coordination in Children with Intellectual and Developmental Disability." International Journal of Environmental Research and Public Health 19(24).	Wrong population: General IDD.
Shields, N., et al. (2022). "A 12-week exercise programme has a positive effect on everyday executive function in young people with Down syndrome: a pilot non-randomised controlled trial." J Intellect Disabil Res 66(12): 924-938.	Wrong outcome: Cognitive / Executive function focus.
Silva, V., et al. (2017). "Wii-based exercise program to improve physical fitness, motor proficiency and functional mobility in adults with Down syndrome." J Intellect Disabil Res 61(8): 755-765	Wrong population: Study conducted on adults.

Supplementary Table S3: PRISMA 2020 Checklist

Section and Topic	Item #	Checklist item	Location where item is reported
**TITLE**			
Title	1	Identify the report as a systematic review.	1
**ABSTRACT**			
Abstract	2	See the PRISMA 2020 for Abstracts checklist.	1
**INTRODUCTION**			
Rationale	3	Describe the rationale for the review in the context of existing knowledge.	2-3
Objectives	4	Provide an explicit statement of the objective(s) or question(s) the review addresses.	3
**METHODS**			
Eligibility criteria	5	Specify the inclusion and exclusion criteria for the review and how studies were grouped for the syntheses.	4-6
Information sources	6	Specify all databases, registers, websites, organisations, reference lists and other sources searched or consulted to identify studies. Specify the date when each source was last searched or consulted.	4
Search strategy	7	Present the full search strategies for all databases, registers and websites, including any filters and limits used.	4
Selection process	8	Specify the methods used to decide whether a study met the inclusion criteria of the review, including how many reviewers screened each record and each report retrieved, whether they worked independently, and if applicable, details of automation tools used in the process.	5-6
Data collection process	9	Specify the methods used to collect data from reports, including how many reviewers collected data from each report, whether they worked independently, any processes for obtaining or confirming data from study investigators, and if applicable, details of automation tools used in the process.	5
Data items	10a	List and define all outcomes for which data were sought. Specify whether all results that were compatible with each outcome domain in each study were sought (e.g. for all measures, time points, analyses), and if not, the methods used to decide which results to collect.	5
	10b	List and define all other variables for which data were sought (e.g. participant and intervention characteristics, funding sources). Describe any assumptions made about any missing or unclear information.	5
Study risk of bias assessment	11	Specify the methods used to assess risk of bias in the included studies, including details of the tool(s) used, how many reviewers assessed each study and whether they worked independently, and if applicable, details of automation tools used in the process.	5-6; (table 3)
Effect measures	12	Specify for each outcome the effect measure(s) (e.g. risk ratio, mean difference) used in the synthesis or presentation of results.	Na
Synthesis methods	13a	Describe the processes used to decide which studies were eligible for each synthesis (e.g. tabulating the study intervention characteristics and comparing against the planned groups for each synthesis (item #5)).	6
	13b	Describe any methods required to prepare the data for presentation or synthesis, such as handling of missing summary statistics, or data conversions.	Na
	13c	Describe any methods used to tabulate or visually display results of individual studies and syntheses.	9-12
	13d	Describe any methods used to synthesize results and provide a rationale for the choice(s). If meta-analysis was performed, describe the model(s), method(s) to identify the presence and extent of statistical heterogeneity, and software package(s) used.	6
	13e	Describe any methods used to explore possible causes of heterogeneity among study results (e.g. subgroup analysis, meta-regression).	Na
	13f	Describe any sensitivity analyses conducted to assess robustness of the synthesized results.	Na
Reporting bias assessment	14	Describe any methods used to assess risk of bias due to missing results in a synthesis (arising from reporting biases).	6,12
Certainty assessment	15	Describe any methods used to assess certainty (or confidence) in the body of evidence for an outcome.	6

PRISMA 2020 Checklist

Section and Topic	Item #	Checklist item	Location where item is reported
**RESULTS**			
Study selection	16a	Describe the results of the search and selection process, from the number of records identified in the search to the number of studies included in the review, ideally using a flow diagram.	7 (figure 1)
	16b	Cite studies that might appear to meet the inclusion criteria, but which were excluded, and explain why they were excluded.	7; supplementary Table S2
Study characteristics	17	Cite each included study and present its characteristics.	7-11 (table 2)
Risk of bias in studies	18	Present assessments of risk of bias for each included study.	8 (table 3)
Results of individual studies	19	For all outcomes, present, for each study: (a) summary statistics for each group (where appropriate) and (b) an effect estimate and its precision (e.g. confidence/credible interval), ideally using structured tables or plots.	9-14
Results of syntheses	20a	For each synthesis, briefly summarise the characteristics and risk of bias among contributing studies.	12-16
	20b	Present results of all statistical syntheses conducted. If meta-analysis was done, present for each the summary estimate and its precision (e.g. confidence/credible interval) and measures of statistical heterogeneity. If comparing groups, describe the direction of the effect.	Na
	20c	Present results of all investigations of possible causes of heterogeneity among study results.	Na
	20d	Present results of all sensitivity analyses conducted to assess the robustness of the synthesized results.	Na
Reporting biases	21	Present assessments of risk of bias due to missing results (arising from reporting biases) for each synthesis assessed.	6,12
Certainty of evidence	22	Present assessments of certainty (or confidence) in the body of evidence for each outcome assessed.	12
**DISCUSSION**			
Discussion	23a	Provide a general interpretation of the results in the context of other evidence.	14-16
	23b	Discuss any limitations of the evidence included in the review.	16-17
	23c	Discuss any limitations of the review processes used.	16-17
	23d	Discuss implications of the results for practice, policy, and future research.	17-18
**OTHER INFORMATION**			
Registration and protocol	24a	Provide registration information for the review, including register name and registration number, or state that the review was not registered.	4
	24b	Indicate where the review protocol can be accessed, or state that a protocol was not prepared.	4
	24c	Describe and explain any amendments to information provided at registration or in the protocol.	Na
Support	25	Describe sources of financial or non-financial support for the review, and the role of the funders or sponsors in the review.	18
Competing interests	26	Declare any competing interests of review authors.	18
Availability of data, code and other materials	27	Report which of the following are publicly available and where they can be found: template data collection forms; data extracted from included studies; data used for all analyses; analytic code; any other materials used in the review.	Na

From: Page MJ, McKenzie JE, Bossuyt PM, Boutron I, Hoffmann TC, Mulrow CD, et al. The PRISMA 2020 statement: an updated guideline for reporting systematic reviews. BMJ 2021;372:n71.

PRISMA 2020 for Abstracts Checklist

Section and Topic	Item #	Checklist item	Reported (Yes/No)
**TITLE**			
Title	1	Identify the report as a systematic review.	yes
**BACKGROUND**			
Objectives	2	Provide an explicit statement of the main objective(s) or question(s) the review addresses.	yes
**METHODS**			
Eligibility criteria	3	Specify the inclusion and exclusion criteria for the review.	yes
Information sources	4	Specify the information sources (e.g. databases, registers) used to identify studies and the date when each was last searched.	yes
Risk of bias	5	Specify the methods used to assess risk of bias in the included studies.	yes
Synthesis of results	6	Specify the methods used to present and synthesise results.	yes
**RESULTS**			
Included studies	7	Give the total number of included studies and participants and summarise relevant characteristics of studies.	yes
Synthesis of results	8	Present results for main outcomes, preferably indicating the number of included studies and participants for each. If meta-analysis was done, report the summary estimate and confidence/credible interval. If comparing groups, indicate the direction of the effect (i.e. which group is favoured).	yes
**DISCUSSION**			
Limitations of evidence	9	Provide a brief summary of the limitations of the evidence included in the review (e.g. study risk of bias, inconsistency and imprecision).	yes
Interpretation	10	Provide a general interpretation of the results and important implications.	yes
**OTHER**			
Funding	11	Specify the primary source of funding for the review.	no
Registration	12	Provide the register name and registration number.	yes

From: Page MJ, McKenzie JE, Bossuyt PM, Boutron I, Hoffmann TC, Mulrow CD, et al. The PRISMA 2020 statement: an updated guideline for reporting systematic reviews. BMJ 2021;372:n71. doi: 10.1136/bmj.n71. This work is licensed under CC BY 4.0. To view a copy of this license, visit https://creativecommons.org/licenses/by/4.0/
